# Massive iris cyst in an adult female

**DOI:** 10.1016/j.ajoc.2025.102278

**Published:** 2025-02-15

**Authors:** Kelvin H. Du, Jennifer J. Bu, Bryanna J. Lee, Kari Hird, Vanessa Goodwill, Nathan L. Scott

**Affiliations:** a Viterbi Family Department of Ophthalmology at the Shiley Eye Institute, University of California San Diego, San Diego, CA, USA; b Department of Pathology, University of California San Diego, San Diego, CA, USA

## Abstract

**Purpose:**

To report an interesting case of a massive iris cyst encompassing nearly the entire anterior chamber in an adult female years after cataract surgery.

**Observation:**

A 77-year-old female with a history of bilateral cataract surgery presented with a large ciliary body mass in the left eye. The lesion was first incidentally noted one year prior to presentation and continued to grow and ultimately caused complete obstruction of the visual axis. Per report, the patient received two YAG laser treatments with subsequent recrudescence. Exam on second opinion consultation with our service was significant for a cystic mass involving nearly the entire left anterior chamber with total occlusion of the pupil. Patient underwent surgical excision with ethanol irrigation and partial iridectomy. The pathology report revealed benign stratified non-keratinizing squamous epithelium consistent with benign epithelial cyst and favoring iris stromal cyst. Post-operative course was complicated by corneal edema and elevated intraocular pressure.

**Conclusion and importance:**

We describe a case of iris implantation cyst that is unique in several aspects, including the size of the mass encompassing nearly the entire anterior chamber, its presentation in an adult years after cataract surgery, rapid growth across one year, and the severe degree of visual compromise.

## 1 Introduction

Iris cysts are epithelial-lined cavities that include a layer of the iris. Broadly, iris cysts are categorized as either primary when there is no identifiable cause, or secondary when there is a known etiology such as surgical or nonsurgical trauma.^1^ Iris cysts are further categorized as pigmented if derived from the iris pigment epithelium or non-pigmented if originating from the iris stroma.^2^ While primary iris cysts are generally more innocuous and often requires no intervention, secondary iris cysts may be more aggressive and require treatment as a result. Implantation cysts, a form of non-pigmented secondary iris cysts, occur after a seeding event, such as penetrating trauma or preceding ocular surgery, followed by proliferation of implanted cells.^2^ In a large case series of 45,500 patients, Terry and associates noted a rate of epithelization of 0.06 % after penetrating ocular trauma or surgical trauma.^3^^,^^4^ Despite being relatively rare, implantation cysts may increase in size, leading to iritis, uveitis, secondary glaucoma, and rarely, occlusion of the visual axis.^2^^,^^5^^,^^6^ Interestingly, the time interval between trauma and clinical presentation of an implant cyst may be as long as 20 years, highlighting the importance of careful history taking in confirming an uncommon diagnosis.^7^ Here, we present a case of a massive secondary implantation iris cyst in a patient with a history significant only for cataract surgery 9 years prior.

## 2 Case report

A 77-year-old female with a history of bilateral cataract surgery presents to an ocular oncology clinic with referral for a possible ciliary body mass in the left eye. Per history, a cyst was noted incidentally, a year prior to presentation, at an annual exam. At that time, she was asymptomatic. The cyst subsequently grew and obscured her vision, after which the patient underwent two Nd:YAG laser procedures, with subsequent recrudescence. Ocular history is significant for asteroid hyalosis in the right eye, blepharitis in both eyes and bilateral cataract surgery nine years prior to presentation. Family history is significant only for a history of ocular melanoma in the patient's father. Visual acuity was 20/25 in the right eye and hand motion on the left. On slit lamp exam, there was a large, cystic mass encompassing nearly the entire left anterior chamber with posterior displacement of the iris and complete obstruction of the pupil. The clear corneal cataract incision was noted at 2:00. Ultrasound biomicroscopy was significant for a massive cyst encompassing nearly the entire anterior chamber.

The patient underwent absolute ethanol irrigation followed by careful visodissection of the cyst along its borders (around the endothelium, iris and intraocular lens) and subsequent extraction through a corneal wound. There were areas of focal adhesions to the iris that was manually stripped using intraocular forceps. There was also an area of iris irregularity and disruption with possible necrosis that was excised to reduce the likelihood of persistent cells.

Pathology report was significant for benign stratified non-keratinizing squamous

epithelium consistent with a benign epithelial cyst and favoring iris stromal cyst, though

no goblet cells were identified.

Post-operative course was complicated by corneal inflammation and edema as well as elevated intraocular pressure in the left eye, which normalized with topical aqueous suppression. Visual acuity in the left eye remained at hand motion at 3 months, largely due to the persistence of corneal decompensation.

## 3 Discussion

Secondary implantation cysts are benign epithelial cysts that typically occur after trauma or ocular surgeries. In a review of 108 eyes found to have anterior segment cysts, 7 cysts (6.5 %) were implantation cysts.^8^ While relatively uncommon, implantation cysts have been known to present with symptoms of iritis and uveitis, and rarely, obstruction of the visual axis^5^ or even secondary glaucoma.^2^^,^^6^ Cases of occluded visual axes secondary to implantation cysts have consisted of cysts ranging from 3 mm to 6 mm in size after variable periods of growth.^5^^,^^9^ In our case, we present a massive cyst, spanning nearly the entire anterior chamber, with posterior displacement of the iris and complete obstruction of the pupil.

The pathogenic mechanism of cyst formation begins with infiltration of epithelial cells into the anterior chamber by means of a surgical wound^10^, ^11^, ^12^ or trauma,^13^^,^^14^ followed by formation of a cystic structure, as in our case. Epithelial downgrowth similarly involves the introduction of epithelial cells into the anterior chamber, however, unlike iris implantation cysts, epithelial downgrowth proliferates to form membranes, typically characterized histologically by stratified squamous epithelium, with multiple layers of epithelial cells. The availability of vasculature in the iris provides the highly proliferative epithelial cells favorable conditions to further propagate.^2^ Growth rate variability is the primary determinant of time between insult and initial presentation. While a high majority of epithelial downgrowth tends to present within 1 year post-operatively,^15^ the longest documented interval between intraocular surgery and epithelial downgrowth is thirty two years.^7^ On the other hand, implantation cysts have a more variable interval to first presentation, ranging from three months to twenty years in two different studies.^16^^,^^17^ In one of the previously mentioned studies, Behruzi and Khodadoust demonstrated that out of 102 implantation cysts, approximately half were attributed to penetrating trauma (49.1 %), whereas the remainder was comprised of a variety of intraocular surgeries, such as intracapsular cataract extraction with vitreous loss (29.9 %), intracapsular cataract extraction (10.8 %), extracapsular cataract extraction (4.9 %), and posterior chamber intraocular lens implantation (2.9 %).^16^ The present patient had history of bilateral cataract surgery approximately nine years prior to first identification of the cyst. The pathophysiology of implantation cysts underscores the importance of proper closure during any intraocular surgery, especially cataract surgery, one of the most common surgical procedures performed.^18^

**Fig. 1:**
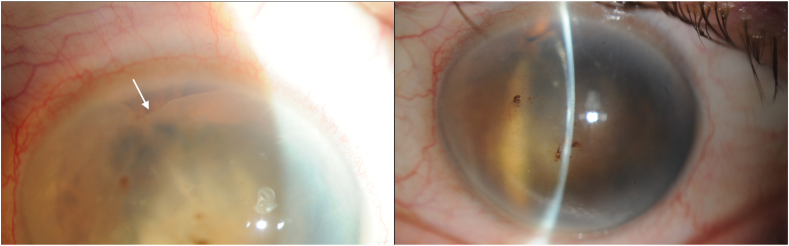
Slit lamp photographs significant for a large cystic mass with posterior displacement of the iris and complete obstruction of the pupil. The white arrow indicates the superior wall of the cyst, which is barely visible in the superior nasal quadrant as the cyst otherwise encompasses the entirety of the anterior chamber.

**Fig. 2:**
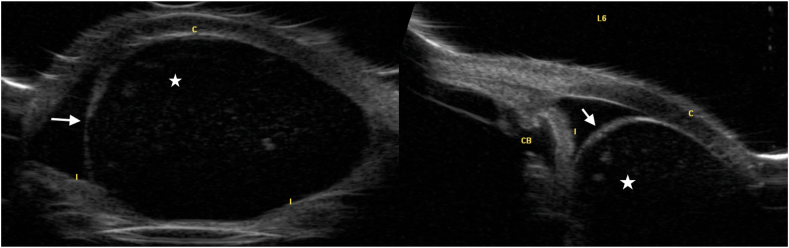
Ultrasound biomicroscopy photographs demonstrating a massive cyst encompassing nearly the entire anterior chamber.

**Fig. 3:**
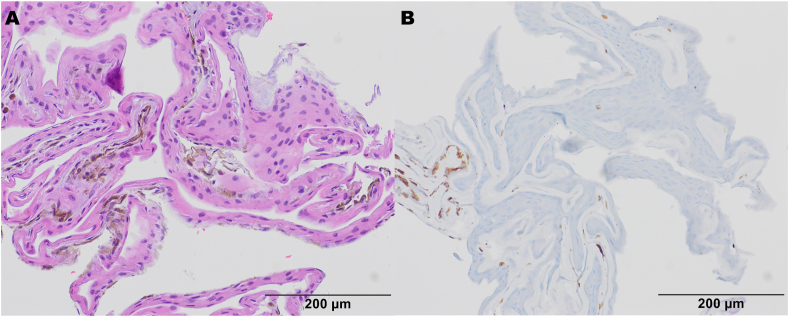
Histopathology of Iris Cyst. (A) H&E of the cell block preparation shows abundant cyst lining composed of non-pigmented stratified squamous epithelium, overlying paucicellular stroma with scattered uveal melanin pigment. (B) S100 and SOX-10 (not shown) immunohistochemical stains were negative, consistent with an iris implantation cyst.

Implantation cysts are diagnosed based on clinical examination, patient history and ultrasound biomicroscopy (UBM) (see Fig. 1). Typically, UBM would be significant for a thick-walled variable-sized cyst with solid or turbid fluid at the iris stroma (Fig. 2).^2^ Despite not being a requirement for diagnosis, histological reports of implantation cysts will demonstrate a lining of concentric layers of stratified squamous epithelium,^2^ as in our current case (Fig. 3a).

Treatment for implantation cysts has typically consisted of an escalating approach based on presence and severity of symptoms. In asymptomatic, stable cysts, observation may be the optimal approach, whereas, symptomatic cysts may be treated with a variety of methods including fine-needle aspiration, intracystic injection of absolute alcohol, mitomycin C, or 5-fluorouracil, laser, and/or lastly surgical manipulation.^2^ While Nd:YAG lasers are less invasive and have been shown to be an effective treatment for symptomatic cysts,^2^^,^^19^, ^20^, ^21^ they have also been shown to be associated with cyst recurrence, as demonstrated in our case as well. Given the evidence of recurrence following Nd:YAG laser treatment, it is possible that the two Nd:YAG laser treatments our patient received contributed to the lesion's accelerated growth. In a long term case series, Gupta and associates noted recurrence in five out of eight cases of post-traumatic iris cysts that were treated with Nd:YAG lasers.^17^ Ethanol irrigation is another efficacious and well-studied procedure for implantation cysts. In a study of epithelial iris cysts treated with ethanol irrigation, Behrouzi and Khadodoust noted 93 out of 102 iris cysts resolved after the first treatment. Similarly, Shields and associates treated 16 cases of iris stromal cysts, 6 of which were secondary, as in our case, and demonstrated involution of cysts in 14 out of 15 cases after ethanol irrigation using a modified technique from the previously mentioned study by Behrouzi and Khadodoust.^22^ In a retrospective study of surgically managed implantation cysts, Shanbhag et al. demonstrated a statistically significantly decrease in recurrence in cases where sector iridectomy was performed in addition to cyst excision, as opposed to cyst excision alone. This suggests that while surgical management may be more invasive, they may also be more effective, especially when less invasive measures such as YAG lasers may lead to recurrence.^23^ Unfortunately, the same study also demonstrated statistically insignificant changes in best corrected visual acuity as a result of associated comorbidities, as in our described case. In a retrospective long-term study of surgically treated epithelial iris cysts, Lan et al. noted that out of eight eyes with corneal edema pre-operatively, six eyes developed post-operative corneal decompensation, requiring penetrating keratoplasty or amniotic membrane transplantation. In addition, Lan and associates also demonstrated postoperative glaucoma found in four eyes post-operatively, managed with trabeculectomies and transscleral diode laser cyclophotocoagulation.^24^ Their postoperative findings further suggest the utility of continued pressure monitoring and longitudinal follow up in these patients.

## 4 Conclusions

We described a massive secondary iris implantation cyst that necessitated ethanol irrigation as well as partial iridectomy. Similar to the previously mentioned studies, our patient developed post-operative corneal edema, intraocular pressure elevation and no improvement in visual acuity. Patients should be counseled on the guarded visual prognoses surrounding this rare diagnosis and when discussing treatment options such as Nd:YAG laser versus invasive surgery. While implantation cysts are a relatively rare long-term post-operative complication of intraocular surgery, the described case highlights the importance of careful closure as a preventive measure, in addition to precise history taking.

## CRediT authorship contribution statement

**Kelvin H. Du:** Writing – review & editing, Writing – original draft. **Jennifer J. Bu:** Writing – review & editing. **Bryanna J. Lee:** Writing – review & editing. **Kari Hird:** Data curation. **Vanessa Goodwill:** Data curation. **Nathan L. Scott:** Writing – review & editing, Conceptualization.

## Patient consent

The patient has given consent to publish this case.

## Disclosures

The authors have no interests to declare.

## Additional contributions

We thank the patient for granting permission to publish this information.

## Authorship

All authors attest that they meet the current ICMJE criteria for Authorship.

## Funding

No funding or grant support

## Declaration of competing interest

The authors declare that they have no known competing financial interests or personal relationships that could have appeared to influence the work reported in this paper.
